# Metabolic adaptation is associated with less weight and fat mass loss in response to low-energy diets

**DOI:** 10.1186/s12986-021-00587-8

**Published:** 2021-06-11

**Authors:** Catia Martins, Jessica Roekenes, Barbara A. Gower, Gary R. Hunter

**Affiliations:** 1 Obesity Research Group, Department of Clinical and Molecular Medicine, Faculty of Medicine and Health Sciences, Norwegian University of Science and Technology (NTNU), Forsyningssenteret, Prinsesse Kristinas Gate 5, 7030 Trondheim, Norway; 2 Centre for Obesity and Innovation (ObeCe), Clinic of Surgery, St. Olav University Hospital, Trondheim, Norway; 3 Department of Nutrition Sciences, University of Alabama At Birmingham, Birmingham, USA

**Keywords:** Metabolic adaptation, Adaptive thermogenesis, Resting metabolic rate, Weight loss

## Abstract

**Background:**

The practical relevance of metabolic adaptation remains a controversial issue. To the best of our knowledge, no study has properly evaluated the role of metabolic adaptation in modulating weight loss outcomes. Therefore, the aim of this study was to determine the association between metabolic adaptation, at the level of resting metabolic rate (RMR), and weight and fat mass (FM) loss after low-energy diets (LED), after adjusting for dietary adherence and other confounders.

**Methods:**

71 individuals with obesity (BMI: 34.6 ± 3.4 kg/m^2^; age: 45.4 ± 8.2 years; 33 males) were randomized to one of three 1000 kcal/day diets for 8 weeks. Body weight, FM and fat-free mass (FFM) (air displacement plethysmography), RMR (indirect calorimetry) and physical activity level (PAL) (armbands) were measured at baseline and at week 9. Metabolic adaptation at week 9 was defined as measured RMR minus predicted RMR at week 9. An equation to predict RMR was derived from baseline data of all participants that were part of this analysis and included age, sex, FM and FFM as predictors. Dietary adherence was calculated from RMR, PAL and body composition changes. Linear regression was used to assess the potential role of metabolic adaptation in predicting weight and FM loss after adjusting for dietary adherence, average PAL, sex, baseline FM and FFM and randomization group.

**Results:**

Participants lost on average 14 ± 4 kg of body weight (13 ± 3%) and presented with metabolic adaptation (−92 ± 110 kcal/day, *P* < 0.001). Metabolic adaptation was a significant predictor of both weight (β = −0.009, *P* < 0.001) and FM loss (β = −0.008, *P* < 0.001), even after adjusting for confounders (R^2^ = 0.88, 0.93, respectively, *P* < 0.001 for both). On average, an increase in metabolic adaptation of 50 kcal/day was associated with a 0.5 kg lower weight and FM loss in response to the LED.

**Conclusion:**

In individuals with obesity, metabolic adaptation at the level of RMR is associated with less weight and FM loss in response to LED.

*Trial registration ID*: NCT02944253.

## Background

Metabolic adaptation, a reduction in energy expenditure below predicted levels in response to weight loss, has been one of the most controversial issues in the Obesity field, both in terms of its real existence, as well as clinical relevance [1–7]. Recent findings from our group suggest that metabolic adaptation depends greatly on the energy balance (EB) status of the participants [8]. Despite being significant during negative EB (on average 100 kcal/d), metabolic adaptation is of minor magnitude (on average 50 kcal/day) when measurements are done under conditions of weight stability following weight loss [8, 9]. This is in line with previous findings showing that the existence or not of metabolic adaptation depends on study design, with cross-sectional studies, comparing obese-reduced individuals to body mass index (BMI)-matched controls, not reporting metabolic adaptation [10–14], while longitudinal studies report metabolic adaptation [15–20], likely because measurements are taken during negative EB.

Despite the abundance of studies, discussed above, on the existence and magnitude of metabolic adaptation, less is known regarding its clinical relevance. Even though several studies have suggested that metabolic adaptation could be a potential explanatory mechanism for resistance to weight loss and/or an important driver of long-term weight regain (relapse) [1–5], no study has, to our knowledge, shown metabolic adaptation to be associated with less weight loss or more weight regain. In fact, we [8, 9] and others [18] have reported metabolic adaptation, at the level of RMR, not to be a risk factor for weight regain. However, we have also recently shown in premenopausal women with overweight, that metabolic adaptation with a 16% weight loss increases the length of time necessary to achieve weight loss goals [21]. Taken together, these results suggest that metabolic adaptation during negative EB may be relevant to weight loss success.

However, it remains to be investigated if metabolic adaptation is associated with worse weight loss outcomes. Therefore, the primary aim of the present analysis was to determine if metabolic adaptation, at level of RMR, was associated with weight and/or fat mass (FM) loss, after adjusting for dietary adherence and other confounders, in a population of individuals with obesity. We hypothesized that metabolic adaptation would be associated with less weight and FM loss in response to low-energy diets (LED).

## Methods

### Participants

Participants in this analysis are part of a large weight loss study (ASKED – Ketosis and Appetite Suppression), aiming to identify the maximum carbohydrate (CHO) intake still associated with appetite suppression in a LED.

The original study enrolled adult (18–65 years old) healthy volunteers, men and women, with obesity (BMI ≥ 30 kg/m^2^), weight stable (< 2 kg variation in weight within the last 3 months), not currently dieting to lose weight and not using any medications known to affect body weight, appetite or metabolism. Given that both RMR and appetite of normally ovulating women has been shown to vary across the menstrual cycle [22, 23], but not in those who take oral contraception [24], we only included post-menopausal women, women taking oral contraceptives or those with a normal menstrual cycle (28 ± 2 days) in the study. This was done to ensure that all measurements were taken in the same phase of the menstrual cycle.

The study was approved by the local ethical committee (REK Midt-Norge, Norway) and all participants provided informed consent before participation. The trial was registered on the 25 *October 2016,* in Clinicaltrials.gov (NCT02944253), https://clinicaltrials.gov/ct2/show/NCT02944253.

### Study design

**Fig. 1:**
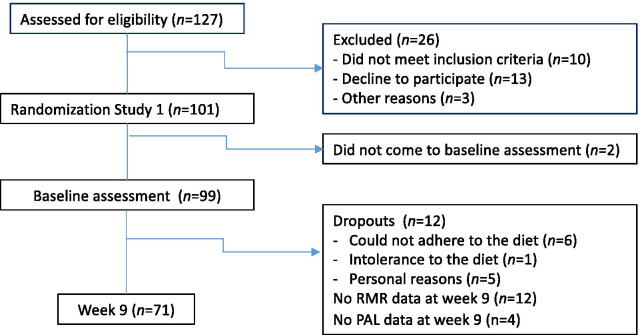
Flowchart of the study. RMR: resting metabolic rate, PAL: physical activity level

This weight loss study was a randomized control trial with repeated measurements. All participants, both men and women, were randomized to one of three isocaloric 1000 kcal/day LEDs containing varying amounts of CHO (70, 100, and 130 g CHO in each group) and a fixed amount of protein (75 g/day). Participants were provided with modular products built specifically for this study (Food Innovation, Oslo, Norway), including shakes and soups, and were encouraged to consume up to 100 g/day of low-starch vegetables and were allowed ad libitum consumption of non-caloric beverages. Participants followed this diet for 8 weeks at the Regional Center for Obesity Research and Innovation (ObeCe) in Trondheim, Norway. For more details about the intervention, please see Martins et al. 2020 [8]. A flowchart of the study can be seen in Fig. 1.

### Data collection

#### Measures of compliance

##### Diet

For the entire duration of the trial, participants attended weekly individual 20-min consultations with study personnel to monitor food intake, body weight (SECA 877 body weight scale, SECA, Hamburg, Germany) and ketosis in urine (acetoacetic acid concentration in urine measured using Ketostix reagent strips (Ascensia Diabetes Care, Basel, Switzerland) and blood (β-hydroxybutyrate concentration in blood measured using FreeStyle Precision Neo (Abbott Laboratories, Quebec, Canada)).

Participants were asked to fill out and submit paper-based food diaries every week detailing all they had consumed each day and to note any side effects they might have experienced. Weekly food diaries obtained from all participants at weeks 2, 5 and 8 of the study were plotted into an online diet planner, Kostholdsplanleggeren (Norwegian Directorate of Health and the Norwegian Food Safety Authority, Oslo, Norway) to estimate actual daily average energy and macronutrient intake during the weight loss phase of the trial.

##### Physical activity

Participants were asked not to change their physical activity (PA) levels during the study. To check for compliance, participants were asked to wear armbands (SenseWear) for 7 days at baseline (week before the start of the study) and at week 8. These data were considered valid if the participants wore the device for ≥ 4 days, including at least one weekend day, more than 95% of the time [25]. Information on physical activity level (PAL) was extracted from the armbands and provided an indication of an individual’s daily physical activity (< 1.40 indicates extremely inactive, 1.40–1.69 indicates sedentary, 1.70–1.99 indicates moderately active, 2.00–2.40, indicates vigorously active and > 2.40 indicates extremely active).

### Outcome variables

The following measurements were conducted at baseline and week 9 (W9) (immediately after weight loss, in negative energy balance), while the participants were in the fasting state and immediately after they had voided in the morning:

#### Body weight, fat mass and fat-free mass

Body weight and composition (fat mass (kg) and fat-free mass (kg)) were determined by whole body air displacement plethysmography (BOD POD, COSMED, Albano Laziale, Italy).

#### Resting metabolic rate

RMR was measured by indirect calorimetry (Vmax Encore 29 N; Care Fusion, Baesweiler, Germany) using a canopy system and standard operating procedures [26].

#### Adherence to diet

Dietary adherence was estimated using a modified version of the original protocol proposed by del Corral and colleagues [27]. First, total energy expenditure (TEE)) was calculated for each individual participant using two methods: (1) RMR x PAL derived from armbands and (2) RMR × 1.4. The results from these two methods were averaged to derive one TEE value for each individual. The reason for this approach is that individuals are likely to increase their PA levels when they wear monitoring devices [28]. TEE at baseline and week 9 was calculated (as described above) and the average of both (TEE baseline and TEE week 9) used. The expected daily kilocalorie loss was calculated as average TEE (baseline and week 9 for each individual)—actual daily average energy provided by the diet (food diaries). Second, to convert losses of FM and FFM (from BOD POD) to energy (*i.e*. kilocalories lost), we used energy coefficients of 9.3 and 1.1 kcal/g, respectively [29, 30]: actual daily kilocalorie loss = [FM lost (g) × 9.3 kcal/g] + [FFM lost (kg) × 1.1 kcal/g]. Third, knowing the actual daily kilocalorie loss and the expected daily kilocalorie loss, we calculated the daily kilocalorie discrepancy, an index of dietary adherence: daily kilocalorie discrepancy = actual daily kilocalorie loss—expected daily kilocalorie loss. A daily kilocalorie discrepancy of zero represents 100% adherence. A positive number would indicate a greater than expected daily kilocalorie loss, whereas a negative number would suggest less than expected daily kilocalorie loss. Fourth, we expressed dietary adherence in relative terms: percent daily kilocalorie adherence = (actual daily kilocalorie loss/expected daily kilocalorie loss) × 100.

### Statistical analysis

Only participants with data available at baseline and W9 were included in this analysis.Statistical analysis was performed with SPSS version 22 (SPSS Inc., Chicago, IL), data presented as mean ± SD and statistical significance set at *P* < 0.05. Changes in body weight and composition over time were assessed with paired t-tests. The presence of metabolic adaptation was tested by paired t-tests, comparing measured RMR (RMRm) and predicted RMR (RMRp) at the same time points. An equation to predict RMR was derived from baseline data of all participants that were part of this this analysis and included age, sex, FM and FFM as predictors.

$$\begin{aligned} & {\text{RMRp }}\left({{\text{kcal}}/{\text{d}}} \right) \, = 505.945 + \left[110.894 \times {\text{sex }}\left(1\,{\text{for}}\,{\text{females}}\,{\text{and}}\, 2\,{\text{for}}\,{\text{males}} \right) \right] \\ & \quad + \left[ 0.402 \times {\text{Age }}\left({{\text{years}}} \right) \right] + \, \left[5.616 \times {\text{FM }}\left({{\text{kg}}} \right) \right] \, + \, \left[ 15.213 \times {\text{FFM }}\left({{\text{kg}}} \right) \right]. \\ \end{aligned}$$

$$R^{{2}} = 0.{79};\;P < \, 0.00{1}$$

Linear Regression models were used to estimate the relationship between weight and FM loss (in kg) at week 9 and metabolic adaptation, after adjusting for diet group (70, 100 and 130 g CHO/day, as a categorical variable) and varaiables known to modulate weight and FM loss outcomes, such as adherence to the diet [27], average PAL [31], sex and baseline body composition (FM and FFM in kg) [32].

Additional analysis, including interaction terms for age, sex and BMI, was performed by introducing an interaction between metabolic adaptation and each of the variables in model, in addition to the other variables (metabolic adaptation, age, sex, BMI, carbohydrate group, PAL, dietary adherence and FFM). FM had to be removed from this model, as FM and BMI are highly correlated and, therefore, their regression coefficients might cancel each other out (multicollinearity).

## Results

Seventy-one adult participants (38 males, 54%) with obesity were included in the present analysis, with an average age of 45 ± 8 years and an average BMI of 35 ± 3 kg/m^2^ at baseline (see Table 1).

**Table 1:** Participant characteristics at baseline and week 9

	Baseline	Week 9	*P* value
Age (years)	45.4 ± 8.2		
Weight (kg)	104.0 ± 14.6	90.1 ± 11.6	< 0.001
BMI (kg/m^2^)	34.6 ± 3.4	30.3 ± 3.0	< 0.001
FM (kg)	43.3 ± 9.1	32.4 ± 8.6	< 0.001
FFM (kg)	60.0 ± 10.9	57.6 ± 9.9	< 0.001
PAL	1.50 ± 0.16	1.48 ± 0.14	0.369
RMRm (kcal/day)	1856 ± 249	1654 ± 204	< 0.001
RMRp (kcal/day)	1856 ± 221	1746 ± 193	< 0.001
RMRm-p (kcal/day)	−0.01 ± 113	*−91.5* ± 110.4***	

Data presented as mean ± SD. P value for the comparison between baseline and week 9. BMI: body mass index, FM: fat mass, FFM: fat-free mass, PAL: physical activity level, RMR: resting metabolic rate. ***P < 0.001 for the comparison between RMRm and RMRp

Participants lost, on average 14.1 ± 0.4 kg of body weight (13.2 ± 2.8%). FM and FFM (kg) were both significantly reduced at W9 compared with baseline (43.3 ± 9.1 vs 32.4 ± 8.6 kg and 60.0 ± 10.9 vs 58.3 ± 10.1 kg, respectively, P < 0.001 for both comparisons). RMRm was significantly lower than RMRp after weight loss (W9), resulting in a metabolic adaptation of -91.5 ± 110.4 kcal/day (*P* < 0.001) (see Table 1).

Metabolic adaptation after weight loss was a significant predictor of both weight (β = −0.009, *P* < 0.001) and FM loss (β = −0.008, *P* < 0.001), even after adjusting for dietary adherence, average PAL, and other confounders (R^2^ adjusted = 0.88 and 0.93, respectively, *P* < 0.001 for both) (see Table 2).

**Table 2:** Regression models for predicting weight and fat mass loss at week 9

Model	B	R^2^ adjusted	*P*
Weight loss at week 9		88%	< 0.001
Intercept	21.669		< 0.001
Metabolic adaptation	−0.009		< 0.001
Adherence to the diet	−0.069		< 0.001
Average PAL	−6.141		< 0.001
Sex	−0.304		0.711
FM at baseline	−0.093		< 0.001
FFM at baseline	−0.277		< 0.001
CHO group	1.490		< 0.001
Fat mass loss at week 9		93%	< 0.001
Intercept	19.275		< 0.001
Metabolic adaptation	−0.008		< 0.001
Adherence to the diet	−0.079		< 0.001
Average PAL	−6.023		< 0.001
Sex	−1.932		< 0.001
FM at baseline	−0.088		< 0.001
FFM at baseline	−0.120		< 0.001
CHO group	1.111		< 0.001

Sex: 1 females; 2 males. PAL: physical activity level; CHO: carbohydrate; FM: fat mass; FFM: fat-free mass

The interaction between metabolic adaptation and age, sex and baseline BMI were not significant either in the model to predict weight or fat mass loss.

## Discussion

The present findings represent the first study examining if metabolic adaptation, at the level of RMR, is associated with the magnitude of weight and FM loss in response to LED. We found that the larger the metabolic adaptation (RMRm-RMRp) in absolute terms, the smaller the weight and FM loss seen, independently of age, sex and BMI, and after adjusting for variables known to modulate weight loss responses, namely adherence to the diet, average PAL, sex and baseline body composition. This suggests that metabolic adaptation may worsen weight loss outcomes during LEDs.

In the present analysis, individuals with obesity who had lost an average of 14 ± 4 kg (13%) of body weight, over 8 weeks on a LED, presented with a metabolic adaptation of approximately −90 kcal/day at week 9. Our regression model showed that even after adjusting for adherence to the diet, average PAL, sex, baseline body composition, and randomisation group, metabolic adaptation was still a significant predictor of both weight and FM loss. On average, for each 50 kcal/day increase in metabolic adaptation, weight and FM loss were reduced by 0.5 kg. This might not seem of clinical relevance, given that the average metabolic adaptation was only approximately −90 kcal/day at week 9. However, in face of the large inter-individual variation in metabolic adaptation seen in the present analysis, ranging from −337 to + 352 kcal/day, that would mean that those with the largest metabolic adaptation (RMRm-RMRp = −337 kcal/day) would lose 3 kg less of body weight and 2.7 kg of FM, compared with those with no metabolic adaptation (RMRm-RMRp = 0 kcal/day). This probably helps to explain some of the variation in weight (−28 to −7 kg) and FM (−19 to −6) loss seen in response to the LED.

It needs to be taken into consideration that we have only looked at metabolic adaptation at the level of RMR and several studies have shown that metabolic adaptation might in fact be of a larger magnitude at the level of non-resting energy expenditure [15, 16]. This suggests that overall, metabolic adaptation (regarding TEE) might have an even larger contribution to weight and FM loss in response to LEDs.

From our knowledge only one study has previously reported an association between metabolic adaptation and weight loss outcomes in response to energy restricted diets. Goele and colleagues [33] reported that metabolic adaptation, at the level of RMR, explained 38% of the difference between measured and predicted weight loss in 22 out of the 48 women with overweight and obesity who experience metabolic adaptation after a 1000 kcal/day diet [33]. However, this study suffers from several important methodological limitations. First, the association was only seen in a subgroup who experienced metabolic adaptation. Second, metabolic adaptation was defined as a reduction in RMR/ kg of FFM. Despite FFM being the main determinant of RMR, FM also contributes to RMR and should be included in the prediction model. Third, no adjustments were done for dietary adherence or PAL of the participants, both important determinants of weight loss outcomes in response to dietary interventions. The present findings confirm the preliminary findings by Goele and colleagues [33] and expand them further, by showing that metabolic adaptation modulates weight loss outcomes in both men and women with obesity, even after adjusting for dietary adherence and other important confounders. Moreover, we have recently shown that metabolic adaptation, at the level of RMR, increases the length of time necessary to achieve weight loss goals (BMI ≤ 25 kg/m^2^), in premenopausal women with overweight [21].

The evidence previously discussed, together with the present findings, suggest that metabolic adaptation might be of clinical relevance. Despite the lack of association between metabolic adaptation and increased risk for weight regain [8, 9, 18], this phenomenon seems to be of clinical relevance in modulating weight loss in the short-term, in response to lifestyle interventions. Clinicians need to be aware of inter-individual variations in metabolic adaptation in response to negative EB when evaluating success to weight loss interventions and not assume that differences between measured and predict outcomes result only from “cheating” (reduced compliance to the intervention).

Our study has both strengths and limitations. The main strength is the fact that we have adjusted for important variables known to modulate weight loss outcomes following energy restricted diets, namely dietary adherence [27], PAL [31], as well as sex and body composition at baseline [32]. Second, this analysis includes a heterogeneous sample of both males and females with obesity, with a wide range of BMI (30–43 kg/m^2^) and age (26–62 years), which is important for generalization purposes. The main limitation of our study is the fact that our estimation of dietary adherence was based on the assumption that energy needs equal RMR x PAL, which is not 100% acurate. However, the error associated with not having taken into account exercise economy is likely minor, as the SD of exercise economy in sedentary individuals has been shown to be around 12% of the mean value [34], meaning that in the present study differences in economy would only account for an average difference of 40 kcal/day in the estimated TEE. Future studies should use TEE data from doubly labeled water to provide a more accurate estimate of energy needs.

## Conclusion

In conclusion, in individuals with obesity, metabolic adaptation at the level of RMR during a 13% weight reduction is associated with less weight and FM loss in response to LEDs. This effect is independent of potential confounders, namely sex and initial body composition, as well as adherence to diet and PAL.

## Data Availability

The datasets used and/or analysed during the current study are available from the corresponding author on reasonable request,pending approval by the local Ethics Committee.

## Abbreviations

BMI: Body mass index
CHO: Carbohydrates
EB: Energy balance
FM: Fat mass
FFM: Fat-free mass
LED: Low-energy diet
PAL: Physical activiy level
RMR: Resting metabolic rate
RMRm: RMR measured
RMRp: RMR predicted
TEE: Total energy expenditure
